# Engineering of Amygdalin Biosynthesis in Rice Endosperm for Pharmaceutical Production and *Sitophilus oryzae* Resistance

**DOI:** 10.1111/pbi.70668

**Published:** 2026-04-21

**Authors:** Ke Chen, Nan Chai, Shaotong Chen, Chanjuan Ye, Kangli Sun, Jie Guo, Xinqiao Zhou, Dagang Chen, Juan Liu, Yanduan Hu, Yi Zou, Rui Cao, Shu Jiang, Bocai Tang, Qinlong Zhu, Chuanguang Liu, Jiantao Tan

**Affiliations:** ^1^ Rice Research Institute, Guangdong Academy of Agricultural Sciences Guangzhou China; ^2^ College of Agriculture, South China Agricultural University Guangzhou China

1

Amygdalin, a cyanogenic β‐glycoside from Rosaceae kernels, has been applied to alleviate cough and asthma, and exhibits anti‐tumour potential (He et al. 2020). A recent study has highlighted its antioxidant, anti‐inflammatory and cardioprotective effects against arsenic trioxide‐induced injury, as well as its anti‐aging properties (Guo et al. 2025). As a natural plant defence metabolite, it is hydrolyzed by animal β‐glucosidases to release toxic hydrogen cyanide, deterring herbivores (Gleadow and Møller 2014). Rice weevil ( *Sitophilus oryzae* ) is a major storage pest of rice, causing excessive yield losses exceeding 20% in developing countries (Nithya et al. 2025). Here, we aim to engineer amygdalin‐rich rice that serves both as a bioreactor for officinal amygdalin production and a novel pest‐resistant agent, which presents considerable potential for the biomanufacturing, medicinal and agricultural applications of amygdalin.

To engineer amygdalin biosynthesis in rice endosperm, we screened candidate enzymes from almond and Japanese apricot (Thodberg et al. 2018; Yamaguchi et al. 2014). The conversion of phenylalanine to amygdalin occurs via two redox reactions and two glycosylation reactions (Figure 1a). For rice expression, the *PdCYP79D16*, isoenzymes *PdCYP71AN24* and *PmCYP71AN24*, iso‐UDP glycosyltransferases (UGTs) *PdUGT94AF3* and *PdUGT85A19*, and diglucosyltransferases *PdUGT94AF2* and *PdUGT94AF1* were codon‐optimized (Table S1). Recombinant *rPdCYP79D16* and *rPdCYP71AN24* were linked using the F2A peptide to generate a single open reading frame (ORF). Likewise, *rPdUGT94AF3* and *rPdUGT94AF2* were fused via P2A peptide. They were placed under endosperm‐specific P_GluB4_ or P_GluB1_ promoters and inserted into a *Cre*/*loxP*‐mediated marker auto‐elimination vector, producing constructs ***C***, ***CC***, ***CCU*** and ***CCUU***, and three variants (***CC'UU***, ***CCUU'***, ***CCU'U′***) with isozyme substitutions (Figure 1b). All constructs were introduced into NanGuiZhan (NGZ), producing T_0_ lines. Transgene insertion and expression were subsequently verified (Figure S1a,b).

**FIGURE 1:**
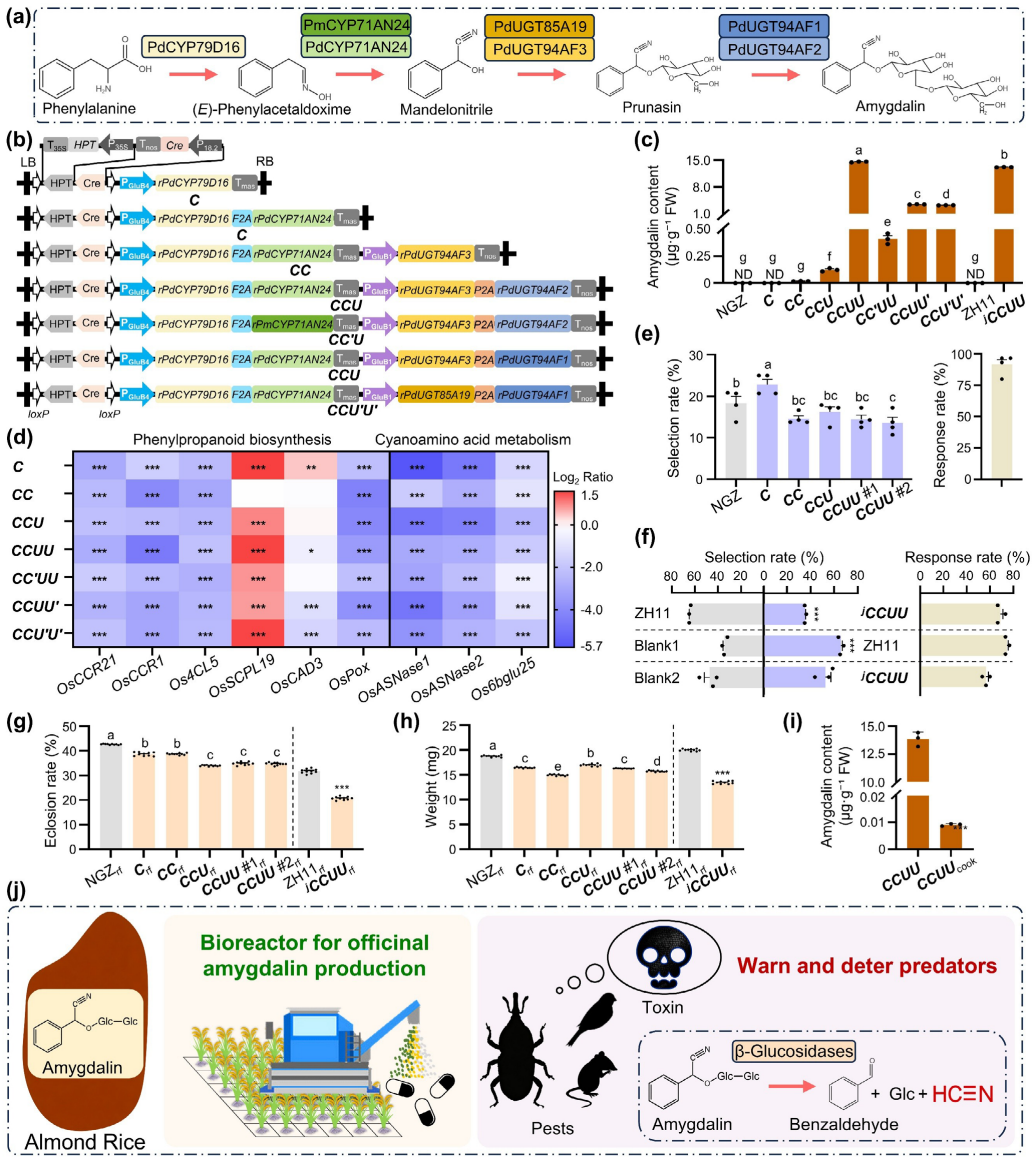
Development of Almond Rice for biomanufacturing and pest resistance via amygdalin metabolic engineering. (a) Key enzymes in the amygdalin biosynthetic pathway. (b) Schematic of the construct design for endosperm‐specific amygdalin biosynthesis in rice. (c) Quantification of amygdalin in transgenic rice endosperm. Bars represent means (± SE, *n* = 3); values sharing the same letter are not significantly different according to Duncan's multiple‐range test (*p* < 0.05). ND, not detectable. (d) Heat map showing the differential expression of endogenous genes in transgenic grains, normalized to NGZ. Statistical significance was determined by Students' *t*‐test (*p < 0.05; **p < 0.01; ***p < 0.001). (e–f) Proportions of rice weevils choosing wild‐type versus transgenic grains (± SE, *n* = 4 in e; *n* = 3 in f). (g–h) Effects on the rice weevil eclosion rate (g), adult development stage (i), and weight (h) after feeding on wild‐type rice (NGZ_rf_ and ZH11_rf_) or transgenic rice (± SE, *n* = 10). (i) Amygdalin levels in *CCUU* and cooked *CCUU* grains (*CCUU*_cook_). (j) Proposed application model for officinal and pest‐resistant Almond Rice.

We used HPLC to measure major metabolites in NGZ and transgenic grains (Figure S2). Phenylalanine was detected in all tested lines. By contrast, (*E*)‐phenylacetaldoxime was undetectable, most likely because *E*‐aldoxime undergoes rapid glucosylation in vivo (Thodberg et al. 2018). Lines expressing *rPdCYP79D16* and *rCYP71AN24s* accumulated mandelonitrile (28.73–88.19 ng·g^−1^), prunasin (0.22–1.83 μg·g^−1^), and amygdalin (0.02–14.54 μg·g^−1^). ***CCUU*** showed the highest amygdalin level (average 14.54 μg·g^−1^), followed by ***CCUU'*** (3.41 μg·g^−1^) and ***CCU'U′*** (3.22 μg·g^−1^). The use of low‐activity PmCYP71AN24 isozyme led to a significantly reduced amygdalin level in ***CC'UU*** (0.41 μg·g^−1^), suggesting that mandelonitrile biosynthesis constitutes the rate‐limiting step for amygdalin production. Low but detectable amygdalin was also observed in ***CC*** (0.02 μg·g^−1^) and ***CCU*** (0.12 μg·g^−1^) lines (Figure 1c; Table S2), potentially reflecting endogenous rice UGTs that act on structurally similar substrates. To test genotype effects, we introduced ***CCUU*** into ZhongHua11 (ZH11), generating ^***j***^ ***CCUU*** grains with 13.17‐μg·g^−1^ amygdalin, slightly below ***CCUU*** (Figure 1c), implying genotype‐dependent yield variation.

Transgenic lines exhibited variable changes in plant height and tiller number, with no consistent trend. No significant differences were observed in other major agronomic traits (Figures S3 and S4). RNA‐seq analysis identified 157 common differentially expressed genes (DEGs) between transgenic lines and NGZ, which were enriched in KEGG pathways including plant–pathogen interaction, phenylpropanoid biosynthesis, and cyanoamino acid metabolism (Figure S5a–c). qRT‐PCR validation confirmed downregulation of lignin‐ and flavonoid‐related genes (*OsCCRs*, *Os4CL5*, *OsCAD3*) and genes involved in cyanoamino acid metabolism and carbon/nitrogen recycling (*OsASNases*, *Os6bglu25*) (Figure 1d; Figure S5d), implying that heterologous amygdalin biosynthesis may disrupt endogenous carbon/nitrogen homeostasis.

Secondary metabolites protect plants by toxic effects, inhibiting feeding, lowering digestive efficiency, or slowing insect growth (Fan et al. 2026; Kortbeek et al. 2019). Since ***CCUU*** and ^***j***^ ***CCUU*** grains accumulated the most amygdalin, we referred to them as Almond Rice and identified homozygous marker‐free lines (Figure S6a–c). We posited that Almond Rice would confer anti‐rice weevil activity. To evaluate pest resistance, we conducted a six‐choice feeding assay using NGZ and multiple T_3_ marker‐free lines, including ***C***, ***CC***, ***CCU***, and two independent ***CCUU*** lines (Figure S7a). When NGZ and transgenic brown rice were placed together, rice weevils preferred NGZ and ***C***, whereas they avoided ***CCU*** and ***CCUU*** grains with higher amygdalin (Figure 1e; Table S3). We then used a Y‐tube olfactometer to assess behavioural responses, with one arm receiving the test odour and the other serving as a control (Figure S7b–c). Rice weevils preferred ZH11 over ^***j***^ ***CCUU*** and clean air (blank control) but showed no preference between ^***j***^ ***CCUU*** and the blank control (Figure 1f; Table S4). We also micro‐inoculated eggs into individual grains, collected emerged adults, and measured traits. Eclosion averaged 42.60% in NGZ_rf_ but declined in ***CCU***_rf_, ***CCUU***_rf_ #1 and #2 (33.98%, 34.76%, and 34.64%) (Figure 1g). Compared with controls, individuals reared on Almond Rice were shorter as adults and lighter, consistent with their smaller body size (Figure 1h; Figure S8a–b; Table S5); similar results were observed for ZH11_rf_ vs. ^***j***^ ***CCUU***_rf_ (Table S6), confirming the pest‐resistant potential of Almond Rice. This resistance may be attributed to the inhibition of antioxidant enzyme activities in rice weevil that consumed Almond Rice (Figure S9).

In addition, we found that amygdalin is unstable at high temperatures: its content in ***CCUU*** grains decreased sharply from 13.83 μg·g^−1^ to < 0.01 μg·g^−1^ after cooking (***CCUU***_cook_) (Figure 1i). Therefore, high‐temperature heating should be avoided during amygdalin extraction.

In this study, we engineered amygdalin biosynthesis in rice endosperm to generate Almond Rice, which displayed improved resistance to rice weevil through reduced feeding preference and growth inhibition. We propose the following application model for Almond Rice: (i) Almond Rice serves as a bioreactor for the industrial extraction of amygdalin. Notably, under strict supervision, Almond Rice may have the potential to replace bitter almond (a traditional Chinese medicinal herb) for its officinal effects. (ii) Almond Rice could be developed into a novel pest‐resistant agent, as it is capable of warning and deterring predators (Figure 1j). Overall, our findings demonstrate the potential of Almond Rice for application in the amygdalin industry, providing a successful example of polygenic bioengineering for crop improvement and biomanufacturing.

## Author Contributions

Conceptualization, J.T. and K.C.; Methodology, K.C., N.C., S.C., C.Y., K.S., Y.H., Y.Z., R.C., S.J. and B.T.; Investigation, J.G., X.Z., D.C. and J.L.; Writing – Original Draft, J.T. and K.C.; Writing – Review and Editing, Q.Z. and J.T.; Funding Acquisition, Q.Z., C.L., J.T. and K.C.; Resources, Q.Z. and C.L.; Supervision, J.T.

## Funding

The work was supported by the Special Funding for the Construction of the High‐Level Academy of Agricultural Sciences (NYQS202628), Special Foundation for Scientific Talents of GDAAS (R2021YJ‐YB3015, R2022YJ‐YB1003, R2022PY‐QY001, R2026PY‐TJ002), the Elite Rice Plan of GDRRI (2025YG02), Youth S&T Talent Support Programme of GDSTA (SKXRC2025524), the Guangdong Key Laboratory of Rice Science and Technology (2023B1212060042).

## Supporting information


Figure S1–S9.
Table S1–S8.

## Data Availability

The authors declare that all data supporting the findings of this study are available within the manuscript and its online Supporting Information S1.
