# Supplementary material for: Engineering of Amygdalin Biosynthesis in Rice Endosperm for Pharmaceutical Production and Sitophilus oryzae Resistance
Source: Plant Biotechnol J. 2026 Apr 21;24(8):4860–2. doi: 10.1111/pbi.70668 (PMC13387877; doi:10.1111/pbi.70668)
Supplement: Supplementary file 1 — Figure S1–S9. Table S1–S8. [file PBI-24-4860-s001.docx]

**Supplemental information**

**Engineering of amygdalin biosynthesis in rice endosperm for pharmaceutical production and *Sitophilus oryzae* resistance**

Ke Chen*, Nan Chai*, Shaotong Chen*, Chanjuan Ye, Kangli Sun, Jie Guo, Xinqiao Zhou, Dagang Chen, Juan Liu, Yanduan Hu, Yi Zou, Rui Cao, Shu Jiang, Bocai Tang, Qinlong Zhu^#^, Chuanguang Liu^#^, Jiantao Tan^#^

**Contents**

**Material and Methods**

**Supplemental Figure 1.** Detection of exogenous genes in transgenic plants.

**Supplemental Figure 2.** HPLC identification of amygdalin and its biosynthetic intermediates in NGZ and transgenic lines.

**Supplemental Figure 3.** Agronomic trait statistics for plant morphology in wild-type (NGZ and ZH11) and transgenic plants (T_3_ lines).

**Supplemental Figure 4.** Grain-related agronomic trait analysis in wild-type (NGZ and ZH11) and transgenic plants (T_3_ lines).

**Supplemental Figure 5.** Identification and validation of differentially expressed genes (DEGs) in NGZ and homozygous T_3_ lines.

**Supplemental Figure 6.** Identification of marker-free Almond Rice in T_3_ populations.

**Supplemental Figure 7.** Behavioural responses of rice weevil to the specific odour of Almond Rice.

**Supplemental Figure 8.** Morphometric characterization of rice weevils reared on wild-type and transgenic rice.

**Supplemental Figure 9.** Analysis of SOD, POD, and CAT activities, as well as MDA levels, in rice weevils.

**Supplemental Table 1.** Nucleotide sequences of codon-optimized *rPdCYP79D16*, *rPdCYP71AN24*, *rPdUGT94AF3*, *rPdUGT94AF2*, *rPmCYP71AN24*, *rPdUGT94AF1*, and *rPdUGT85A19*.

**Supplemental Table 2.** Concentrations of key metabolites in the amygdalin biosynthetic pathway in fresh grains from NGZ, ZH11, and transgenic lines.

**Supplemental Table 3.** Proportions of rice weevils selecting NGZ versus transgenic lines.

**Supplemental Table 4.** Proportions of rice weevils selecting ZH11 versus ***^j^CCUU*** rice.

**Supplemental Table 5.** Developmental metrics measured in rice weevils reared on NGZ and transgenic lines.

**Supplemental Table 6.** Developmental metrics measured in rice weevils reared on ZH11 and ***^j^CCUU*** rice.

**Supplemental Table 7.** Oligos used in this study.

**Supplemental Table 8.** Gene accession numbers used in this study.

**Materials and Methods**

**Rice constructs and transformation**

The coding sequences of *PdCYP79D16*, *PdCYP71AN24*, *PdUGT94AF3*, *PdUGT94AF2*, *PmCYP71AN24*, *PdUGT85A19*, and *PdUGT94AF1* (NCBI accession no. GU573413.1, XM_034360738.1, MH969427.1, MH969428.1, NP_001313440.1, XM_034370835.1, and AYQ98970.1) were codon-optimized for rice expression and chemically synthesized by Tsingke Biotechnology Co., Ltd. (Beijing, China). To generate a fused coding sequence, the recombinant *rPdCYP79D16* and *rPdCYP71AN24* were connected using the F2A peptide with the amino acid sequence GSVKQTLNFDLLKLAGDVESNPGPGS, producing *rPdCYP79D16-F2A-rPdCYP71AN24*. In parallel, the P2A peptide (GSATNFSLLKQAGDVEENPGPGS) was used to link *rPdUGT94AF3* and *rPdUGT94AF2*, yielding the fusion construct *rPdUGT94AF3-P2A-rPdUGT94AF2*. The *rPdCYP79D16* and *rPdCYP79D16-F2A-rPdCYP71AN24* fragments were inserted into the *Mlu*I site of a modified pCambia1300 vector using a modified Gibson assembly strategy (Zhu et al., 2014) and placed under control of the rice endosperm-specific promoter P_GluB4_, generating constructs ***C*** and ***CC***, respectively. The rice endosperm-specific promoter P_GluB1_ was then used to drive *rPdUGT94AF3* and *rPdUGT94AF3-P2A-rPdUGT94AF2*, which were subsequently cloned into the *Asc*I site of construct ***CC*** to produce constructs ***CCU*** and ***CCUU***. To generate isozyme variants, *rPdCYP71AN24*, *rPdUGT94AF2*, and *rPdUGT94AF3* in ***CCUU*** were individually replaced with *rPmCYP71AN24*, *rPdUGT94AF1*, and *rPdUGT85A19*, resulting in constructs ***CC'UU***, ***CCUU'***, and ***CCU'U'***, respectively. All constructs contained a *Cre* recombinase cassette driven by the heat-inducible promoter P_18.2_ (Takahashi et al., 2011), with the *HPT* and *Cre* cassettes flanked by two *loxP* sites to enable excision.

All amygdalin biosynthesis constructs were introduced into the *indica* rice cultivar NanGuiZhan (NGZ) or the *japonica* rice cultivar ZhongHua11 (ZH11) using an *Agrobacterium tumefaciens*-mediated transformation protocol (Nishimura et al., 2006). Transformation procedures were carried out by Wuhan Biorun Co., Inc. Integration of target genes in the resulting transgenic plants was verified by PCR using gene-specific primers (Table S7).

**Plant growth conditions and verification of agronomic traits**

All transgenic lines were grown in paddy fields in Guangzhou (23°39′N, 113°43′E) under natural field conditions. For metabolite extraction, seeds were collected at 30 days after pollination. Agronomic traits of wild types (NGZ and ZH11) and transgenic plants were evaluated at the mature stage.

**Heat-induced excision of marker genes**

To excise the *HPT* and *Cre* cassettes using the heat-inducible *Cre/loxP* system, germinating T_3_ seeds from transgenic lines were exposed to a programmed temperature regimen in a growth chamber: 2.5 h at 42 °C under light (13,000 lux) followed by 9.5 h at 28 °C under light, then 2.5 h at 42 °C in darkness followed by 9.5 h at 28 °C in darkness. After 7 days of treatment, seedlings were transplanted to experimental plots, and genomic DNA was extracted from leaf tissue for PCR verification using the primer pairs P-F/P-R1 and P-F/P-R2 (Table S7).

**Expression analysis and RNA-seq analysis**

Total RNA was extracted from rice seeds collected at 20 days after pollination using TRIzol reagent (Invitrogen, USA) following the manufacturer’s instructions. First-strand cDNA was generated using the TransScript cDNA Synthesis Kit (TransGen Biotech, Beijing, China). Transcript levels of both transgenes and endogenous genes were quantified by qRT-PCR using the following cycling conditions: 95 °C for 3 min, followed by 40 cycles of 95 °C for 10 s, 58 °C for 15 s, and 72 °C for 20 s. *OsActin1* (*LOC_Os03g50885*) served as the internal reference for normalization. Three biological replicates were analyzed, and all primer sequences are listed in Table S7.

RNA-seq was performed by Biomarker Technologies Corporation (Beijing, China). Common differentially expressed genes (DEGs) between transgenic lines and the NGZ control (*P* < 0.05, absolute log_2_FC≥1) were identified and subjected to Kyoto Encyclopedia of Genes and Genomes (KEGG) enrichment analysis using the KEGG pathway database (http://www.genome.jp/kegg/). To confirm the transcriptome results, 17 endogenous genes were used for qRT-PCR validation. Gene accession numbers used in this study are provided in Table S8.

**Metabolite extraction and quantitative analysis**

Amygdalin and its pathway intermediates were extracted from 0.5 g of mature brown rice grain powder under dark conditions by incubation with 5 mL of extraction solvent (0.1% formic acid and 20% methanol solution) at 4 °C for 30 min. Samples were centrifuged at 12,000 rpm for 5 min at 25 °C, after which the supernatant was passed through a 0.22 μm filter membrane and used for high-performance liquid chromatography (HPLC) analysis on an Agilent 1260 LC analyser. The mobile phase consisted of A (0.1% formic acid solution) and B (acetonitrile), and separations were carried out on a Poroshell 120 EC-C18 column (2.7 μm, 100 mm × 2.1 mm) maintained at 40 °C. The gradient program was as follows: 90% A for 0–2 min; linear decrease to 5% A from 2–9 min; and then a linear increase back to 90% A, which was maintained until the end of the run (12 min). The flow rate was set to 0.3 mL·min^–1^. HPLC measurements were conducted by Nanjing Webiolotech Testing Technology Co., Ltd.

The metabolite content was calculated using *ω* = *ρ* × *V* × *m*⁻¹, where *ω* denotes the metabolite content (μg·g^–1^), ρ is the sample concentration (μg·mL^–1^), *V* is the sample volume (mL), and *m* is the sample weight (g). Metabolite standards (phenylalanine, CAS NO: 63-91-2; (*E*)-phenylacetaldoxime, CAS NO: 7028-48-0; mandelonitrile, CAS NO: 532-28-5; prunasin, CAS NO: 99-18-3; amygdalin, CAS NO: 29883-15-6) were serially diluted to generate calibration curves, which were then used to quantify metabolite levels in rice grain samples.

**Rice weevil feeding assay**

Rice weevils were maintained in an incubator at 28 °C and 70% relative humidity under continuous darkness. Newly eclosed adults were randomly chosen for subsequent assays and were deprived of food for 24 h in the dark before testing.

The feeding preference assay was conducted according to Fan et al. (2025). NGZ and different transgenic brown rice samples (approximately 10 g per line) were each placed in separate transparent plastic boxes, and the boxes were positioned evenly within a large circular container. Adult rice weevils were released at the centre of the container, and their feeding was recorded for 10 min in darkness. Four biological replicates were performed, with 30 adults per replicate.

For the specific odour response assay, ZH11 and ***^j^CCUU*** brown rice (approximately 10 g per line) were separately loaded into glass bottles. The airflow system was assembled in the following order: air pump, drying tower containing activated carbon, water tower, glass bottles with rice samples, flowmeters, and a Y-tube olfactometer. Using the flowmeters, airflow was adjusted to 370 mL·min^–1^. Adult rice weevils were introduced at the Y-tube inlet. Individuals that moved upwind and reached the midpoint of either Y arm within 10 min were scored as responsive to that specific odour, and the number of responding adults was recorded. The ZH11 and ***^j^CCUU*** rice samples were then replaced with blank controls, respectively. For each tested group, three biological replicates were conducted, with 30 adults per replicate.

**Measurement of development indicators in rice weevils fed Almond Rice**

Rice weevil eggs were carefully inoculated into individual brown rice grains. The infested grains were incubated under controlled conditions (28 °C, 70% relative humidity) to permit completion of a full generation. Emerged adults were then collected and subjected to comprehensive morphometric analyses to quantify biological traits.

**Antioxidant enzyme activities and malondialdehyde levels measurement**

Adult rice weevils were collected from NGZ and ***CCUU*** rice grains after three days of feeding. For each sample, 50 individuals were prepared, rapidly frozen in liquid nitrogen, and homogenized in 1.0 mL of ice-cold phosphate buffer (0.1 M, pH 7.4) supplemented with 1% polyvinylpolypyrrolidone (PVPP) using a glass homogenizer. Enzyme activities and malondialdehyde (MDA) levels were determined by using enzyme linked immunosorbent assay (ELISA) following the manufacturer's instructions. Commercial kits for measuring superoxide dismutase (SOD), peroxidase (POD), catalase (CAT) activities and MDA levels were purchased from Shanghai Enzyme-linked Biotechnology Co., Ltd. All assays were performed in quadruplicate for each treatment group, and the results were normalized to fresh weight.

**Supplemental Reference**

**Fan, Y., Tang, Y., Miao, Y., Zhao, Y., Yu, L., Han, P. and Zhu, X.** (2025) Developing eco-friendly, pest-resistant cotton by a heterologous multi-gene transformation system for caffeine synthesis. *Crop J*. doi.org/10.1016/j.cj.2025.06.005.

**Nishimura, A., Aichi, I. and Matsuoka, M.** (2006) A protocol for Agrobacterium-mediated transformation in rice. *Nat. Protoc*. **1**, 2796–2802.

**Takahashi, T., Naito, S. and Komeda, Y.** (2011) The Arabidopsis Hsp18.2 Promoter/Gus Gene Fusion in Transgenic *Arabidopsis* Plants - a Powerful Tool for the Isolation of Regulatory Mutants of the Heat-Shock Response. *Plant J*. **2**, 751–761.

**Zhu, Q., Yang, Z., Zhang, Q., Chen, L. and Liu, Y-G.** (2014) Robust multi-type plasmid modifications based on isothermal in vitro recombination. Gene **548**, 39–42.

**
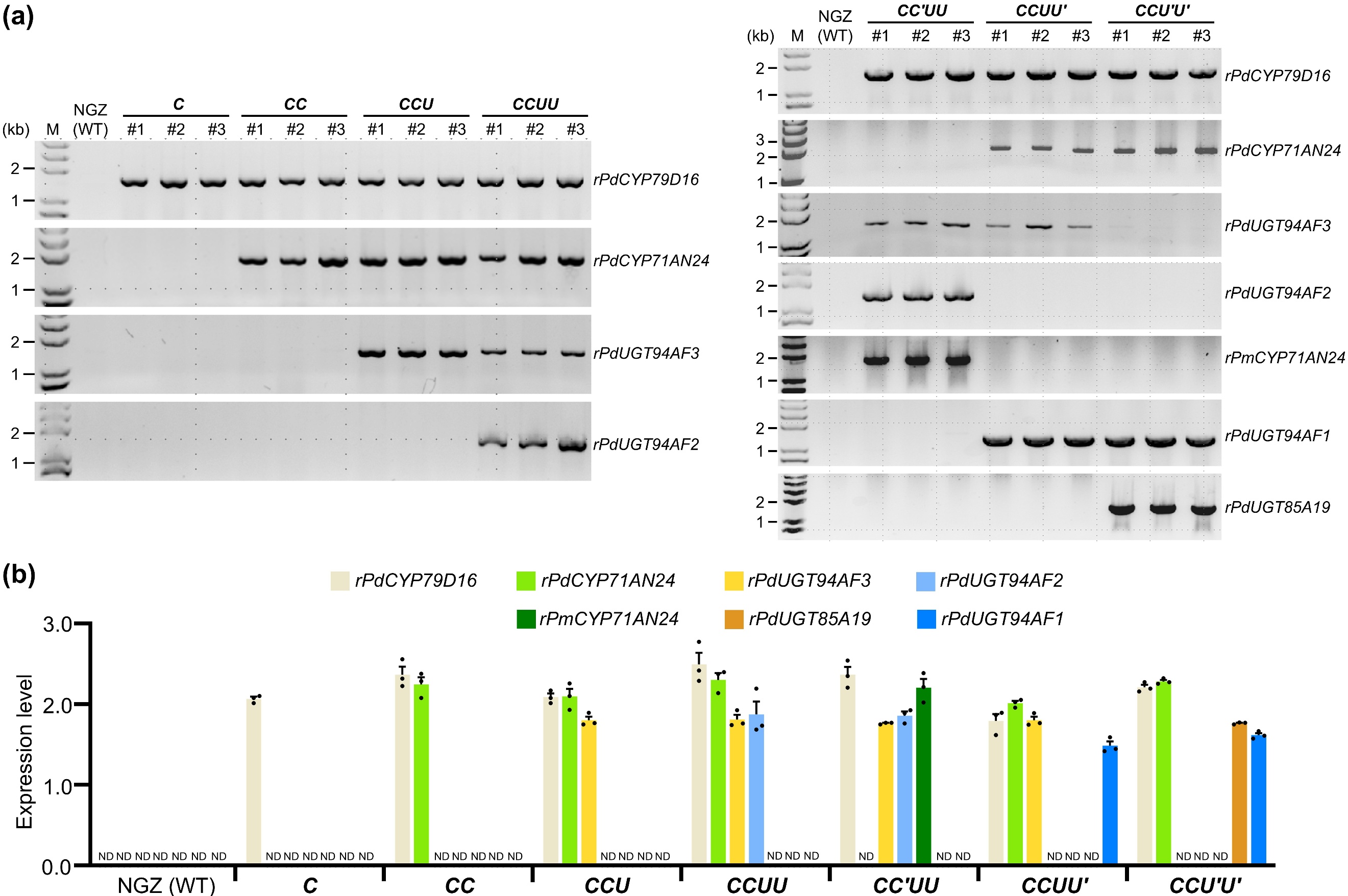
**

**Supplemental Figure 1.** Detection of exogenous genes in transgenic plants.

**(a)** PCR verification of three independent T_0_ lines for each construct (***C***, ***CC***, ***CCU***, ***CCUU***, ***CC'UU***, ***CCUU'*,** and ***CCU'U'***) using specific primers (Supplemental Table 7). NanGuiZhan (NGZ, wild type, WT) served as the negative control. M, marker. **(b)** Expression analysis of transgenes in rice endosperm from NGZ and transgenic plants (homozygous T_3_ lines). Data are shown as mean ± SE from three biological replicates. *OsActin1* was used as a reference.

**
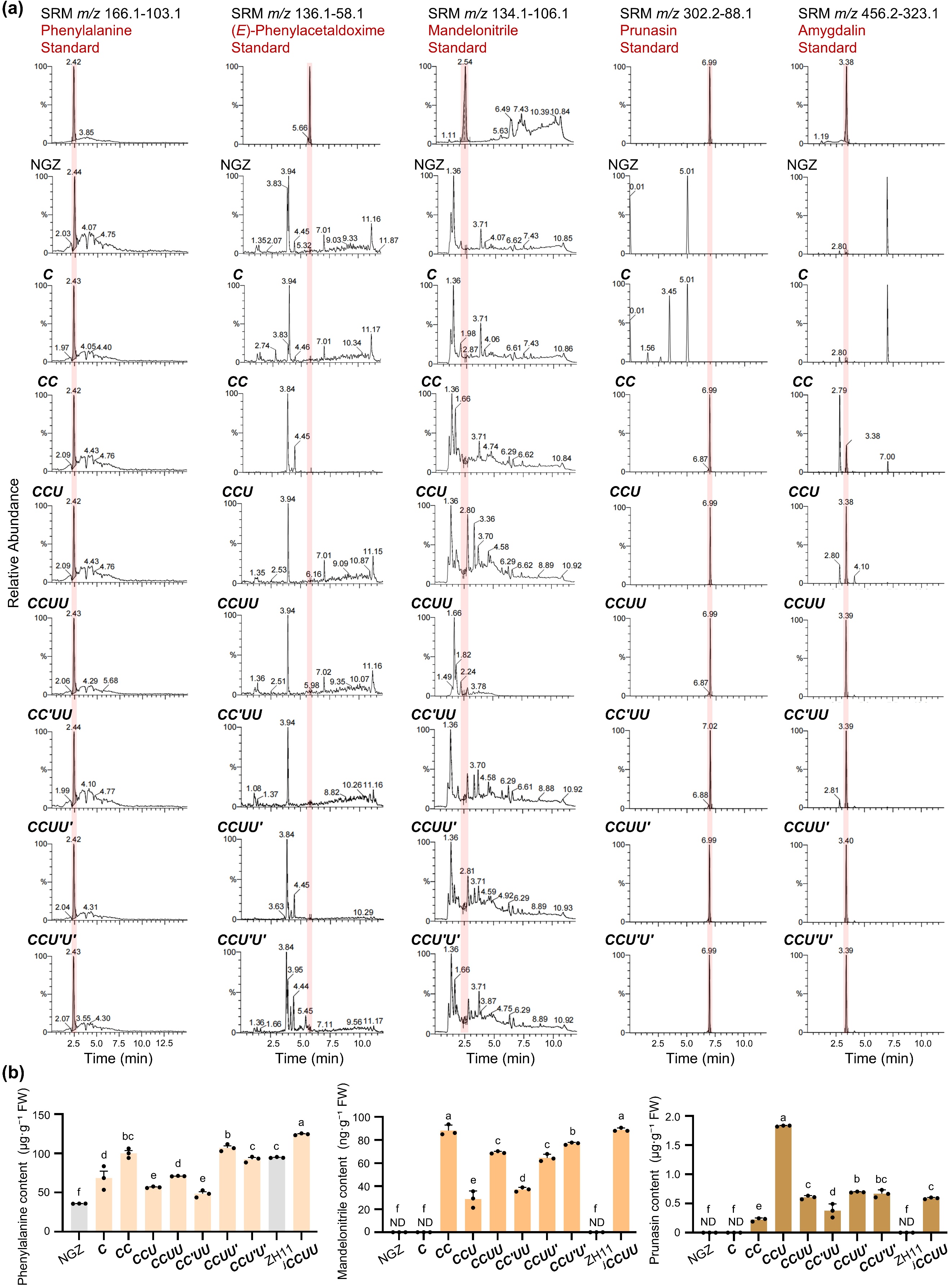
**

**Supplemental Figure 2.** HPLC identification of amygdalin and its biosynthetic intermediates in NGZ and transgenic lines.

**(a)** HPLC chromatograms showing peaks corresponding to phenylalanine, (*E*)-phenylacetaldoxime, mandelonitrile, prunasin, and amygdalin in authentic standards, NGZ, and one representative line from each construct. SRM, selected reaction monitoring. **(b)** Quantification of phenylalanine, mandelonitrile, and prunasin in transgenic rice endosperm. Bars represent means (± SE, *n* = 3); values sharing the same letter are not significantly different according to Duncan's multiple-range test (*P* < 0.05). ND, not detectable.

**
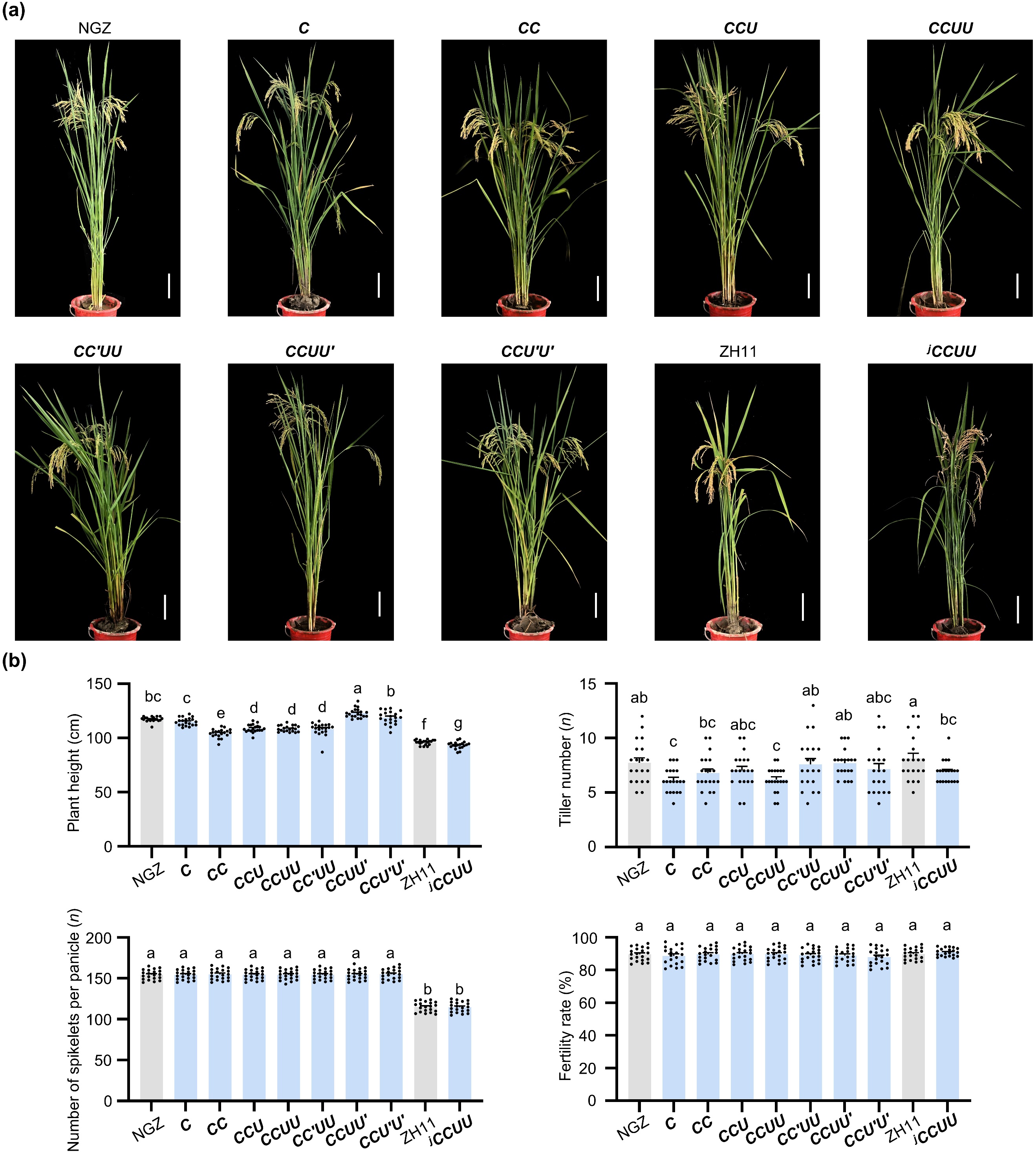
**

**Supplemental Figure 3.** Agronomic trait statistics for plant morphology in wild-type (NGZ and ZH11) and transgenic plants (T_3_ lines).

**(a)** Representative plant morphology of NGZ, ZH11, and transgenic lines. Bars, 5 cm. **(b)** Quantification of plant height, tiller number, spikelets per panicle, and fertility rate. Values are means (± SE, *n* = 20); groups sharing the same lowercase letter are not significantly different according to Duncan's multiple-range test (*P* < 0.05).


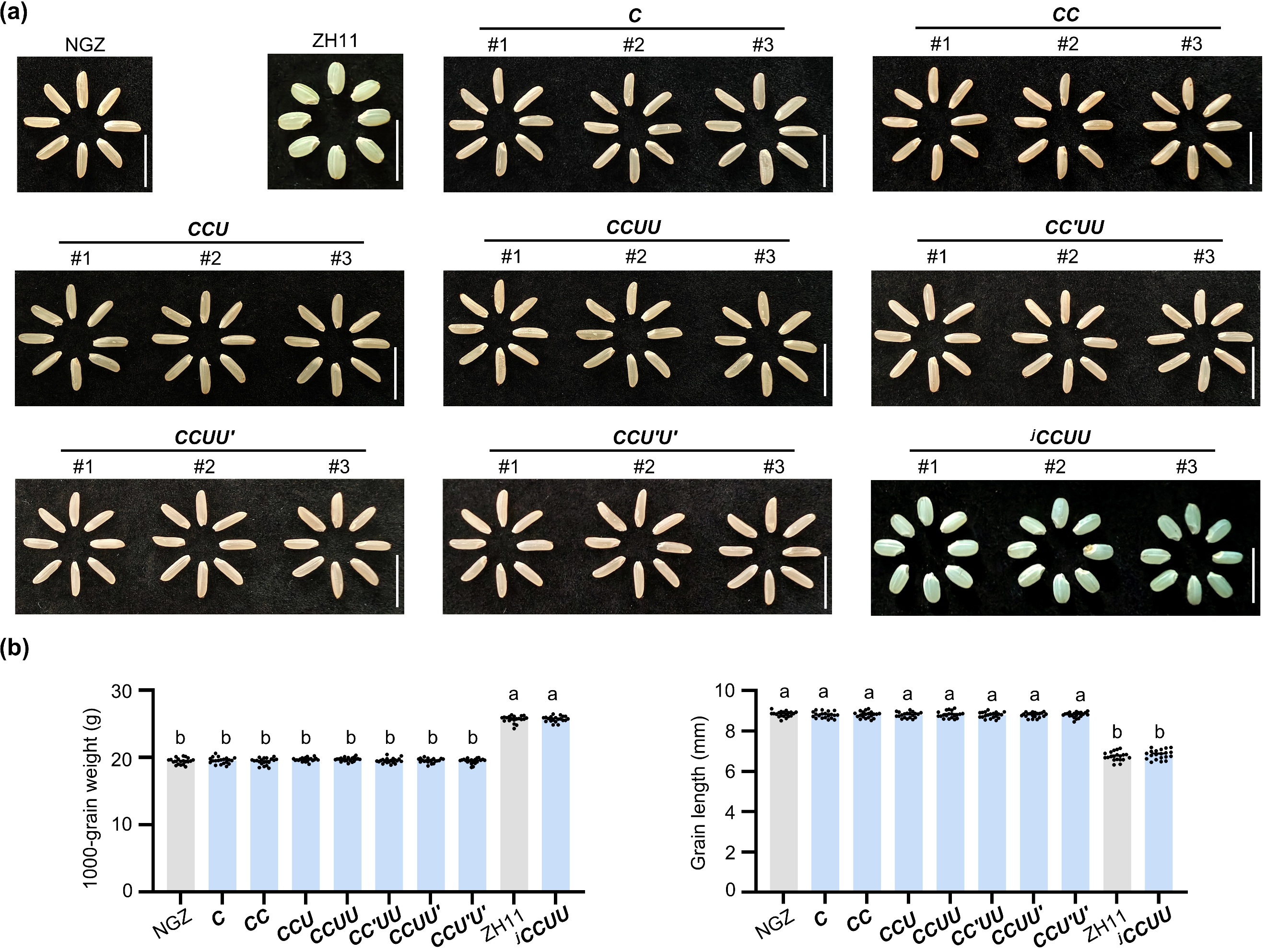


**Supplemental Figure 4.** Grain-related agronomic trait analysis in wild-type (NGZ and ZH11) and transgenic plants (T_3_ lines).

**(a)** Representative brown rice phenotypes from NGZ, ZH11, and transgenic lines. Bars, 1 cm. **(b)** Measurements of 1000-grain weight and grain length. Data are presented as means (± SE, *n* = 20); values with the same lowercase letter are not significantly different based on Duncan's multiple-range test (*P* < 0.05).

**
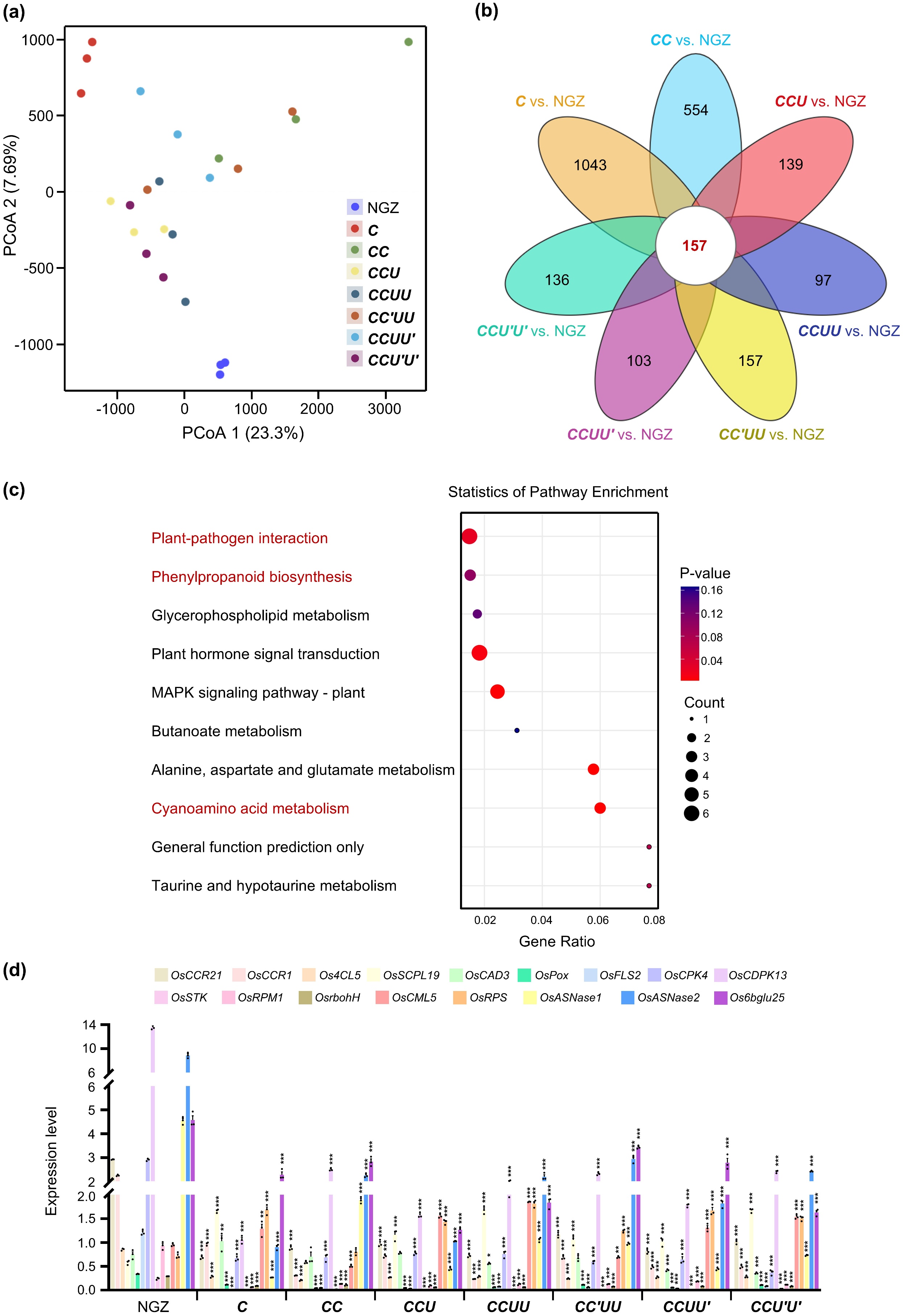
**

**Supplemental Figure 5.** Identification and validation of differentially expressed genes (DEGs) in NGZ and homozygous T_3_ lines.

**(a)** Principal coordinate analysis (PCoA) among the indicated groups. **(b)** Venn diagram depicting DEGs identified between NGZ and each transgenic line; the number in bold red indicates the DEG count in the overlapping region. **(c)** Kyoto Encyclopedia of Genes and Genomes (KEGG) enrichment analysis of DEGs in transgenic lines. Circle size denotes gene number, and colour represents the q-value; key pathways are highlighted in red. **(d)** qRT-PCR validation of selected endogenous DEGs in grains from NGZ and transgenic lines. Data are presented as with means ± SE (*n* = 3; **P* < 0.05, ***P* < 0.01, ****P* < 0.001) based on Students' *t*-test, with *OsActin1* used for normalization.

**
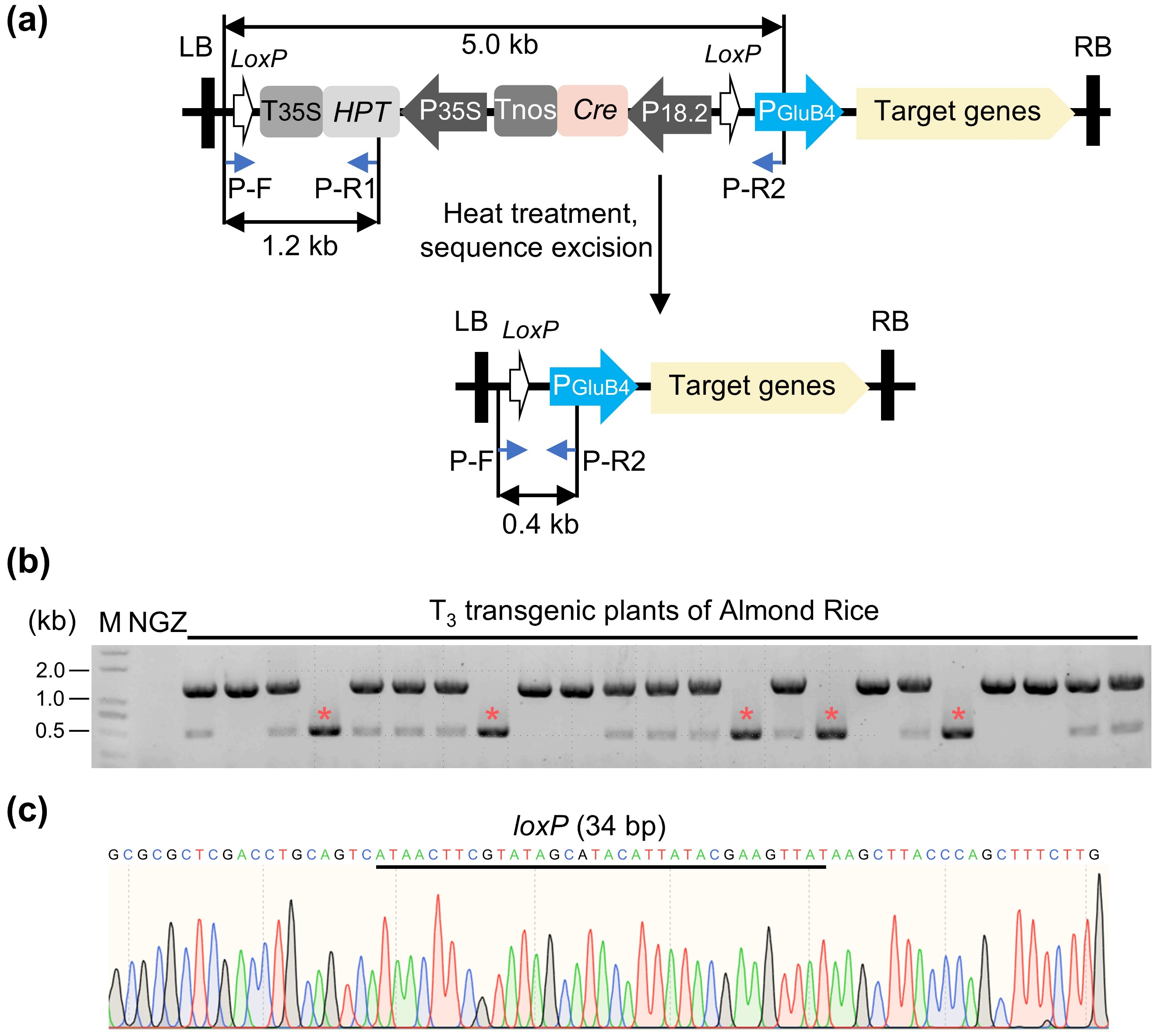
**

**Supplemental Figure 6.** Identification of marker-free Almond Rice in T_3_ populations.

**(a)** Schematic of the transformation constructs and the marker-free strategy. After heat induction, the T-DNA region carrying the marker gene and the *Cre* expression cassette is removed via *Cre*/*loxP*-mediated site-specific recombination. Successful excision yields a 0.4-kb recombined fragment amplified with primers P-F and P-R2, whereas failure to excise produces a 1.2-kb fragment amplified with primers P-F and P-R1. **(b)** PCR detection of marker cassette excision in T_3_ Almond Rice lines using the P-F/P-R1/P-R2 primer set. Homozygous marker-excision plants are indicated by an asterisk. M, marker. **(c)** Sanger sequencing confirmation of the marker-free recombined products.

**
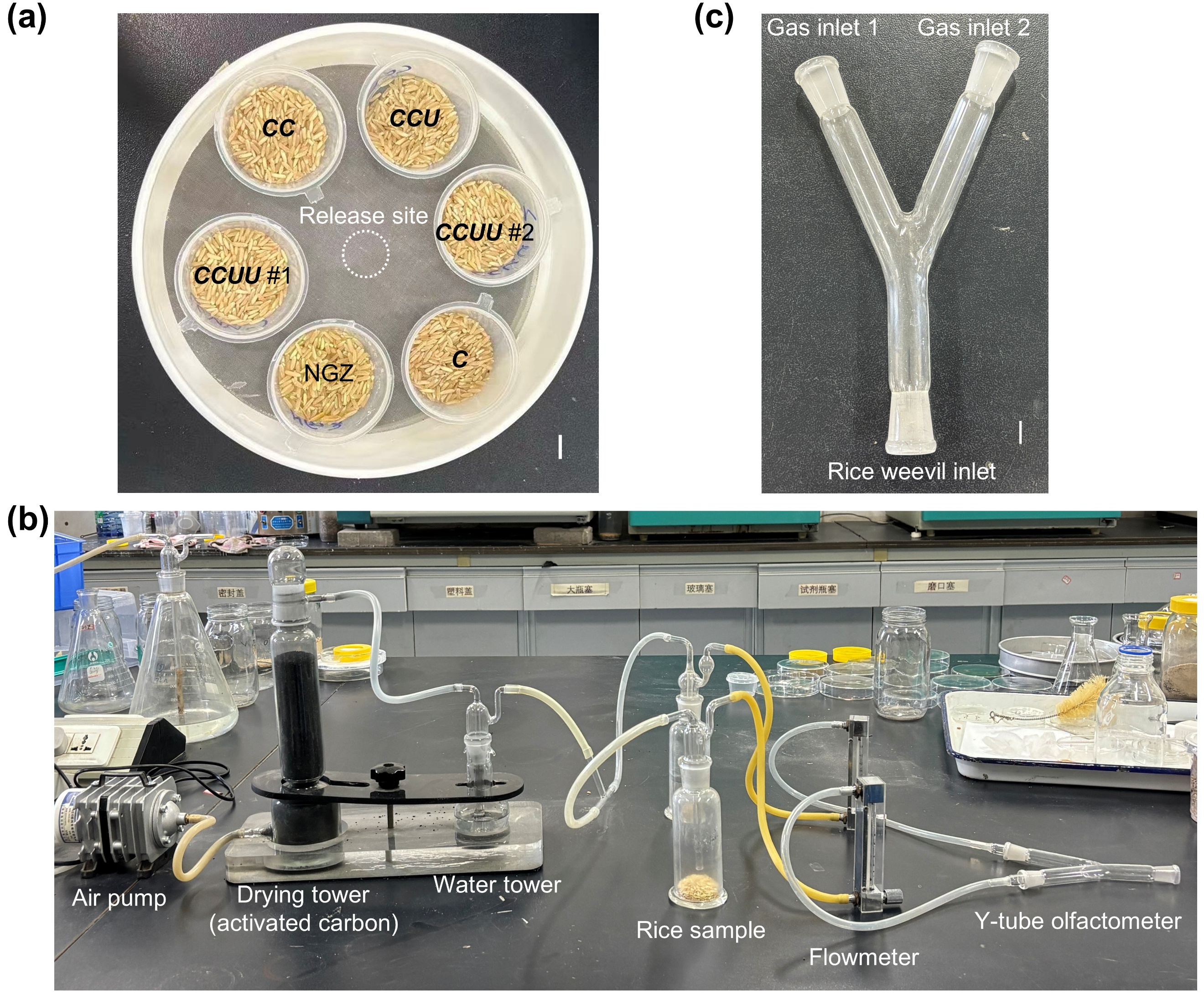
**

**Supplemental Figure 7.** Behavioural responses of rice weevil to the specific odour of Almond Rice.

**(a)** Diagram of the rice weevil feeding-preference assay using NGZ and transgenic grains. Bar, 1 cm. **(b)** Overview of the rice weevil odour preference assay using NGZ or Almond Rice. **(c)** Configuration of the Y-tube olfactometer used in this study. The specific odour from rice grains (ZH11 or ***^j^CCUU*** lines) or a blank control was delivered to one arm of the Y, and rice weevils were released at the inlet. Individuals that moved to the midpoint of either arm were scored as responsive to the corresponding odour. Bar, 1 cm.

**
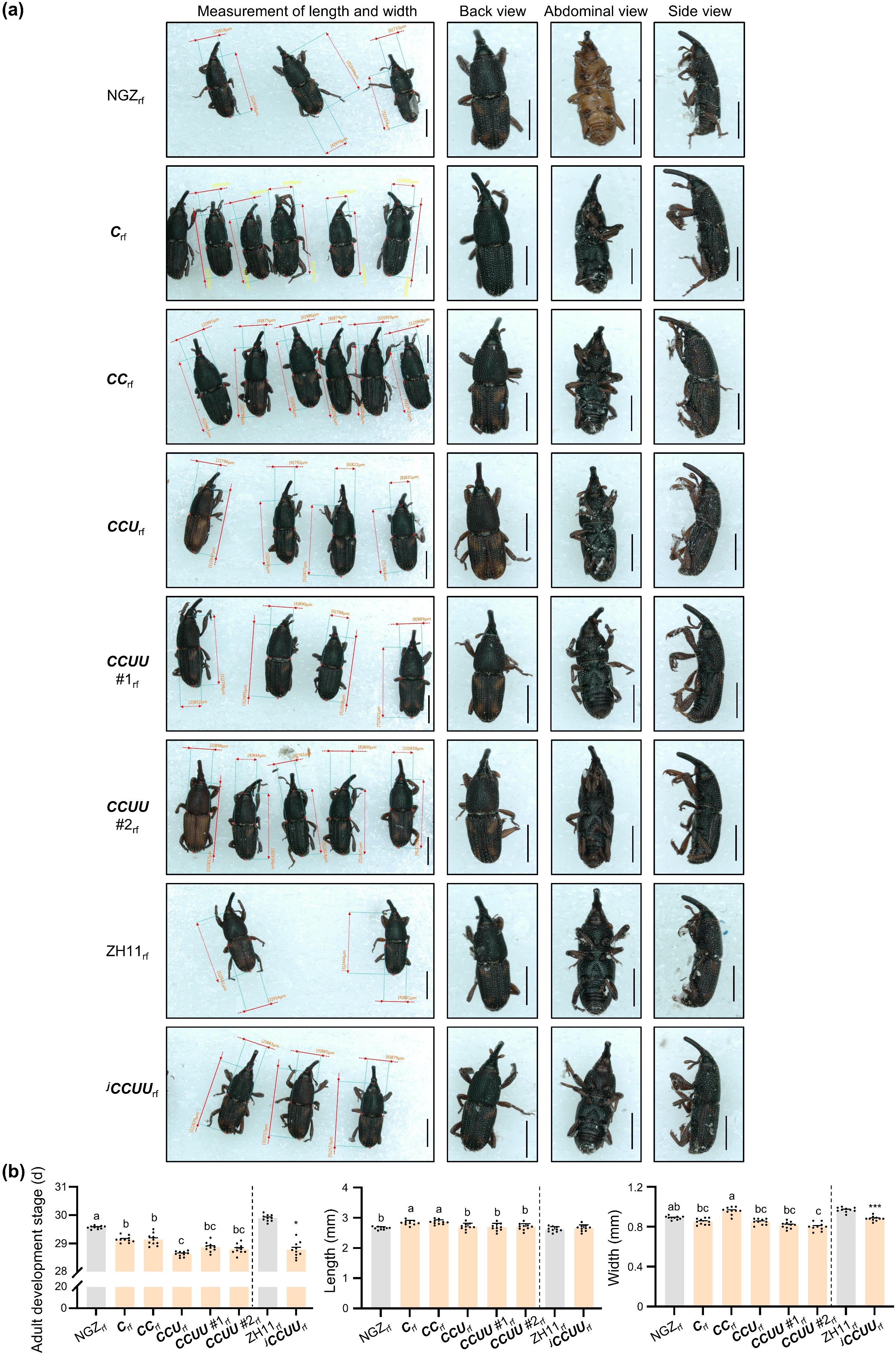
**

**Supplemental Figure 8.** Morphometric characterization of rice weevils reared on wild-type and transgenic rice.

**(a)** Assessment of body length and width in rice weevils fed wild-type rice (NGZ_rf_ and ZH11_rf_) or different transgenic lines. Dorsal, abdominal, and lateral views illustrate representative morphological features. Bars, 1 mm. **(b)** Quantitative analysis of adult development stage, body length and width in NGZ_rf_, ZH11_rf_, and transgenic rice-feeding groups. For NGZ background groups, data are shown as means (± SE, *n* = 10), and groups sharing the same lowercase letter are not significantly different according to Duncan's multiple-range test (*P* < 0.05). For ZH11 background groups, values represent means ± SE (*n* = 10), with significance determined by *t*-test (***, *P* < 0.001).

**
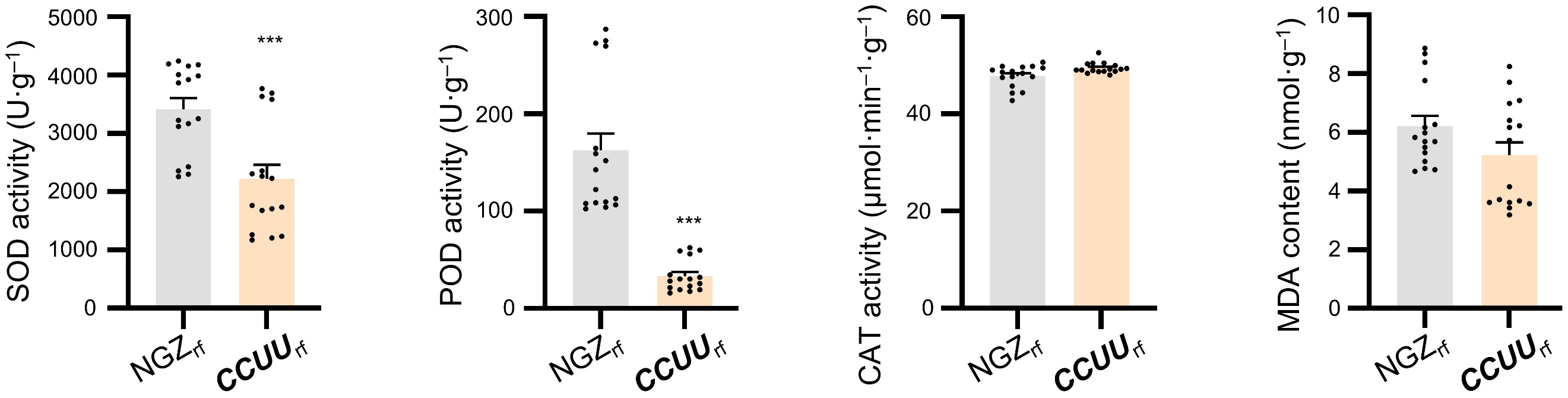
**

**Supplemental Figure 9.** Analysis of SOD, POD, and CAT activities, as well as MDA levels, in rice weevils.

Enzyme linked immunosorbent assay (ELISA) was used to quantify superoxide dismutase (SOD), peroxidase (POD), and catalase (CAT) activities, as well as malondialdehyde (MDA) levels, in rice weevils fed on NGZ (NGZ_rf_) and ***CCUU*** (***CCUU***_rf_) lines. Values represent means ± SE (*n* = 16), with significance determined by *t*-test (***, *P* < 0.001).

**Supplemental Table 1.** Nucleotide sequences of codon-optimized *rPdCYP79D16*, *rPdCYP71AN24*, *rPdUGT94AF3*, *rPdUGT94AF2*, *rPmCYP71AN24*, *rPdUGT94AF1*, and *rPdUGT85A19*.

| Genes | Sequence (5′–3′) |
| --- | --- |
| *rPdCYP79D16-F2A-rPdCYP71AN24* | ATGGAAGCTAACGTGGGCTTTCTCACTCTCTGTCTGGCTATAACACTCGTTAGGTTCCTCATGAAGCGGTACTGGCACCAATCAAAAATTAACGACAATAACAACAAGGCGATCAAGCAGCACTACCCTCTGCCGCCGACCCCTAAAGGACTGCGGCCCTGGCCAATCGTCGGCAACCTGCCCGAAATGCTCATGAATAAACCCACCTTTCGGTGGATACACAAGCTTATGGAGGAGTCTAACACCGAAATAGCGTGCATCCGGCTGGCGAATGTGCATGTTATCCCAGTGAGCTGTCCCATCCTCTCCAGGGAAATCCTCAAAAAGCAGGATGCGACCTTTGCAACAAGACCGCTCTCCATCAGTACCTTCCTCATAACTAAGGGTTATATCACAACAGTTATGGTCCCGTTTGGCGAGCAGTGGAAAAAGATGCGCAAGGTCATCACAAGTGAGCTTCTTTCCCCAATGAGGCATAAGTGGCTCACTGATAAGCGCATCGAAGAAGCGGATCACCTCGTCAGGTACGTCTTCAATCAATGTAACAATGAGGAAGGGCGCGGCATCGTGGACCTGCGCCTTGCCACGCAGCACTACTGCGCGAATGTCATCAAGAGGATGATATTCAACCAGAGGTATTTTACTGAAGAAATGAAGGACGGCGGCCCGTCAGTTGAGGAGCAGAACTACGTGAATGCAGTCTTCGACATGCTGCGGTATATTTACGCTTTCAGCGCGTCTGATTACATTTCATGTCTCCGGGGCCTTGACCTTGACGGGCACGAGAAGATTATAAAGGATTGTATCAAGCTGACAAGAAAGCGCCAGGATCCCGTCATCGAGGAAAGGATCCGGGAGCATCAGAAACTCGGCGGAAATAAAGTCCCTGTTGACCTCTTGGATATCCTTATTTCCCTCAAGGACGCCTCTGGGCAGCCCTTGCTGTCTCCAGATGAGATAAAGGGCCAGGTTAACGAAATGATCATGGCTGCAGTCGATAATCCTTCTAATGCCGCCGAATGGGCTATTGCTGAGATGATAAACCAACCGCACCTTTTTGAGAAGGCGCGGCAAGAGTTGGACGCGGTGGTGGGTAAAGAGAGGCAGGTGCAAGAATCAGACCTGAGCCAGTTGAACTTCGTGAAGGCGTGCGCCCGGGAGGCCTTCCGTCTGCACCCTGTTGCGCCGTTTAACGTACCACACGTGTCCATGGCTGACACAACAGTTGGAGATTACTTTATCCCCAAGGGTTCACACGTTATGCTTTCTAGAATCGGCCTCGGTCGGAACCCAAAGATTTGGGATGAGCCCCTCAAGTATAAGCCCGAGAGGCACTTGAAGGACGATGGGTCTGGTGTGGTCCTCACTGAGAGTGAGCTCAGATTCATAAGCTTTTCGACCGGCATGAGAGGGTGCGTTGCTTCCACCCTCGGTACTTCCATGACCGTGATGCTTTTCGCTAGGCTCCTGCACGGGTTTACCTGGGAAGCCCCTCCGAACGAGAGCAGGATAGACTTGACCGAGGCTGGAGGCGAGCTGCTTCTCGCTAAGCCGCTCTTGGCCCTGGCGAAACCGCGCTTGCCGGCGCACGTGTACCAAACTGGATCTGTGAAGCAGACATTGAACTTTGACTTGCTGAAGCTGGCGGGGGATGTGGAGAGCAACCCGGGGCCTGGCTCTATGGCGCTGTTGACCTTGTTTAATCAGATCTGGCAGGAGGGACAACTCCAGTCCTCGACATCTTCTTTCAACATCTTTCTGGTGCCTATTCTTTTCCTCTCAATCTTCATCCTCTTTTCCCTCACCCGCTCGTCTTCGCCAAGCGAGAAAAACAGGAAGCTTAAGCTCCCACCATCCCCCCCGCGCCTCCCATGGATCGGTAATCTCCATCAGCTGGGTAGTTTTCCGCACCGCTCACTGAGGGCCCTTTCGAAGAAGTATGGCGACGTGATGTTCATGCATTTCGGGAAGGTCCCCACACTCATAGTGTCATCCGCCGAGATGGCTAAGGACGTGATGAAAACGCAGGATATCGTCTTTTGTAGCCGTCCTCAAACCACCGCCCCGTCGATTCTGTTTTACGATGGCCACGACATAGCTTTCGCCCCGTACGGCGAGTACTGGCGCCAGGTCAGGAGGATATGTGTGCTCGAGTTGCTTAGTCTGAAACGCGTGCATCAGTTCCAATACGCTCGCGTGGAGGAAGTAGCTGAGCTGGTCTCGAAGATTAGGAAGGCCTCTGCGTCGGCCAATGGAGCCCCGATCAATCTCGGGGAGCTGTTGGTAAGCACTTCTAATAATATCATATGTCGCTGCATACTCGGGCAAAAGTTCGAAGATAAAGAGGATAACTGGTTCGGCGAAACCACAAAGGAGCTCATGACGCAGGTGATGAGCTTCTCTTTCGGAGATTTCTTTCCGAGTCTGAAGTGGATCGACCGGGCGCGCGGCTATCTGGCCTACCTGAAGTCGATTTGGCTCGAGTTCGACAAGTTTTTCGATAAACTCATTGATGAGCACAAGGCAGCGCAAAAGGAGGGGAAGCCGCGCAAGAAAGACATTGTGGACATACTGCTTGATGTTCAAAAGGATGGCTCGTTGGATTTCGAGCTGACCACCAGCAACGTTAAGGCGATCCTCCAAGATATGTTCGTGGGTGGGAGCGACACGTCCTGGACGGCTGCCATATGGCTTATGAGCGAGCTTAGCCAGAATCCTCGGGTCATGAAGAAGGTCCAGGAGGAAGTCAGAAGGGTAGCTGGCAAACGCGGCTACGTCGAGGAGAGTGACATTAACGAAATGAAATATCTTACCTGCGTCATTAAGGAGAACCTCCGGCTGCACCCTCCGGCTCCTCTGCTGCTGCCTCGTGAGGCCATGAGCGACGTCAAGCTGGGCGGCTTTGACATCCCAGAAAAGACGCAAGTGTTCGTGAACGCCTATGCGGTACAGAGGGATCCAAAAGTGTGGGACAAACCGGATGAATTCATGCCCGAAAGATTTGAGGAGAACAATGTTGGGTTTGTCGGTCAAGACTTTGAGCTGATTCCTTTCGGCGCGGGTCGGAGAGTATGTCCAGGCCTTGCCTTCGGCGTGGCGAGTGCCCAGTATGTGCTCGCGAATATGCTCTACTGGTTCGATTGGAAGCTGCCGTCAGGCGGATCAAAATTGGCCGAGACCCTCGACATGAGCGAGGTGTATGGCCTCACGGTCCACAAGAAGAGCCCACTGTACTTGGTACCTACGCCCTACTCCCCCTGA |
| *rPdUGT94AF3-P2A-rPdUGT94AF2* | ATGGATAGCAGTCAGCAAAGAAAGTTTCGGGTCCTGATGTTCCCGTGGCTGGCCCACGGGCACATTTCTCCCTACCTGGAACTGGCCAAGAAGCTGACAAATCGCAATTTCCACATTTACTTCTGTAGCACCCCGGTCAATCTCAGGTCGATAAAGCCCCAGTTGTCAGAGAAGTACTCCAGGTGCATTGAGCTCGTTCAGTTGCATCTCCCTTATGATGACCTGCCAGAACTGCCGCCTCACTACCACACGACGAATGGCCTCCCGCCCCACCTGATGTCAACATTGAAAACAGCCTTCGATAGGGCCTCCCCTAACTTCTCTAATATTCTGAAAACGCTCCATCCTGATCTCTTGATATACGACTTCCTTCAGCCATGGGCTCCTTCCCTGGCGTTGCTTCAGAACATTCCAGCAATTGAATTTTTCACCACATCGGCGGCTATGATGTCAGTGTGTACCCACCACGGGGAGAAGCCAGGTGTGAAGTTTCCTTTCCCATCAATCTACTATGAGACTAGCAAGATCAAAATGTTGCTCGAGTCCTCCTCTAATGGAATCTCTGATGGCGATAGGGCCAAGCAATGCAGTGACAGGTCGTGTAAAATCGTGCTGGTTAAATCATCCCGGGAGATTGAGGCCAAGTATATTGATTACCTGAGCGATCTCATCGGCAAGAAAATTGTCCCGGTAGGGAGCCTGATCCAGGACCTTATCGAGCAGGAAGTGGATAGCGAAGAGACGAAGATCATGAAGTGGCTGAATACTCGTGAGCGGTCTTCCGTGGTCTATGTTAGCTTCGGATCTGAATACTTCCTCAGCAAAGAGGAGATCGAGGAAATCGCCCACGGTCTTGAACTTTCTAAGGTTTCGTTCATCTGGGTTATCAGGTTTCCGAAAGAGGAGAAAGGAACACGGGTCGAAGAGGTCTTGCCTAAGGGATTCCTGGAGAGGGTGGGAGAGAAAGGGATCATTGTCGATGGTTGGGCCCCGCAAGCGAAGATCCTGAAGCACTCCAGTGCAGGGGGCTTCGTCAGCCATTGCGGATGGTCAAGTGTCCTTGAGAGCATCAAGTTCGGTGTCCCGATTGTCGCGATGCCCATGCACTTGGATCAACCCATCAACGCGAGAATAGTGGAGGACGTGGGGGTTGGAGTTGAAGTCAAGAGAATGGGCGGGGGGGGCAATGAGAACGGAAGACTGAAGCGCGATGAGATCGCTAAGGTCATTCGTGATGTCGTCGTGGAGGAGAACGGTCAGGGCTTGAAACGGAAGGCCATGGAGCTCCGCGATAACATGAAGAAGCGGGAGGACGAAGAGATCGATGGGGTGGTGGAACAGCTCATCCAGCTCTGTATGAGAAAAGAGGGCTCCGCTACTAACTTCTCTCTCTTGAAACAGGCTGGTGACGTTGAAGAGAACCCTGGTCCTGGCTCCATGGTGTATTCCGAGCATAAGAGCATCACTGTGCTGATGCTGCCCTGGTTGGCGCATGGGCATATCTCCCCCTTCCTCGAGCTCGCGAAAAAGCTGACCTCAAAGCGCAACTTCCATATTTTCATATGCTCGACACCGGTGAACCTGACTTCCATCAAGCCGAAGCTGAGTCCAAAGTACTCTCATTGTATAGAGTTTGTCGAACTCCACCTCCCGCACGAAGAGCTCCCCGAGCTTCCACCCCACTACCACACCACCAACGGACTCCCACCGCATCTCATGAGCACACTCAAGAGGGCCTTCGATATGAGCTCGAACAACTTCTCCAACATTCTCACAACACTTAAGCCAGATCTGCTTATATATGACTTCATTCCTCCATGGGCGCCTTCGCTCGCCTCTCTTCAAAACATCCCTTCAGTCAGATTCATTACCACGTCGGCTGCGCTTAGCTCCCTCCGGGTGCACCACCTCAAGAATCCGAGAGTGAAGTTTCCGTTCCCTTCGATTTATCTCAGAGATTATGAGGCCAAGAAGTTCAATAATTTGTTGGAGCCAAGCTCGAATGATATCAACGACGGAGACAGGGTTCAGCAATGCTCGGCACGCTCCTGCAACATCATTCTTGTTAAGACATCACGCGAGATAGAGGCGAAGTATGTGGATTATCTTTCCGGGCTCATGGGGAAGAAGATCGTGCCCGTAGGACCACTCGTCCAGGAACCCATGGATCTCAAGGTCGACGAAGAAACGTGGATTATGGAGTGGCTGAACAAACGCGAAAGGTCCAGTGTCGTCTACGTGTGCTTTGGCTCGGAGTATTTCCTCTCCCGCGAGCAAATCGAGGAGTTGGCGCACGGACTCGAGTTGTCTAAGGTCTCTTTCATATGGGTTATTCGCTTCCCAAAGGAAGAGAAGGGAAAGAGAGTCGAGGAAGTCCTCCCCGAGGGATTCCTGGAGCGTGTCGGCGAAAAAGGCATCATCGTCGAAGGCTGGGCACCACAAGCAAACATTCTCAAACATAGTTCGGTGGGCGGCTTCGTGTCGCATTGTGGGTGGTCGTCGGTTTTGGAGAGTATCAAGTTCGGCGTTCCTATCATCGCTATGCCGATGCATCTCGACCAGCCGATCAACGCCCGCCTCGTTGAGGAGGTGGGCGTTGGGGTCGAAGTGAAACGGACCGGAGAGGGAATCTTGCAGAGGGAGGAAATGGCGAAGGTTATTAGGAAGGTGGTTGTGGAAAAGATAGGCGAGGGCATGCGCAAGAAAGCATTGGAGCTCCGGGATAATATGAAGAACAAGGACGGCGAAATTGATGGTGTAGTTGAGGAACTCATGCAGTTCGTCGGTGAGGGGAAACAAATTAGCACATTTAAGTGA |
| *rPmCYP71AN24* | ATGGCATTGCTCACGCTCTTCAACCAAATCTGGCAGGAGGGACAATTGCAGTCATCAACCTCGAGCTTCAATATATTCTTGGTGCCAATCCTTTGCCTCAGTATCTTCATCCTTTTTAGTTTGACGAGGAGCTCAAGCCCTAGTGAAAAGAACCGGAAGCTGAAATTGCCACCGTCGCCACCGCGGCTCCCTTGGATTGGAAATCTGCATCAACTGGGTTCATTTCCGCACAGATCGCTTCGTGCGCTCTCTAAAAAGTATGGGGATGTGATGTTCATGCACTTCGGCAAGGTCCCCACACTCATCGTCAGCAGCGCAGAGATGGCCAAGGACGTCATGAAAACCCAGGACATTGTCTTTTGCTCACGTCCACAGACGACAGCCCCTAGCATCCTGTTTTATGATGGCCACGACATCGCCTTCGCTCCATACGGGGAGTACTGGCGGCAGGTTCGCAGGATTTGCGTGCTCGAGCTTCTCTCACTTAAACGTGTTCACCAGTTCCAGTACGCCCGCGTGGAGGAGGTCGCCGAGCTGGTCTCCAAGATCCGCAAGGCAAGCGCTTCTGCTAATGGTGCCCCCATAAACCTTGGCGAACTCCTCGTTTCAACGTCGAACAATATTATTTGCAGATGTATCCTTGGCCAGAAATTCGAGGATAAAGAGGATAATTGGTTCGGGGAGACCACCAAGGAACTGATGACGCAGGTAATGAGCTTTAGTTTCGGCGATTTCTTCCCGTCGTTGAAGTGGATCGACAGAGCACGGGGGTACCTCGCCTACCTGAAGTCGATATGGCTTGAATTCGACAAGTTCTTCGACAAACTGATAGACGAGCATAAGGCAGCGCAGAAGGAGGGCAAGCCGCGCAAGAAAGACATAGTGGACATATTGCTCGATGTGCAGAACGACGGCTCTCTTGACTTTGAGCTGACCACATCCAATGTGAAAGCTATATTGCAGGACATGTTTGTCGGCGGTAGCGACACCTCTTGGACAGCGGCCATCTGGCTCATGAGTGAGCTTAGCCAGAACCCTCGCGTTATGAAAAAGGTCCAGGAGGAAGTGAGGAGGGTCGCCGGTAAGCGCGGATATGTCGAGGAGTCCGACATCAACGAGATGAAGTACCTCACATGTGTTATCAAGGAGAATCTCCGGCTGCATCCACCAGCCCCGCTGTTGCTGCCGCGGGAGGCCATGTCTGACGTCAAGCTCGGCGGGTTCGACATCCCGGCAAAGACCCAGGTTTTCGTCAACGCATATGCCGTGCAGCGCGATCCGAAAGTCTGGGATAAGCCGGACGAATTTATGCCAGAGCGGTTTGAGGAAAATAACGTAGGGTTTGTCGGCCAAGATTTCGAGCTCATTCCCTTCGGAGCTGGCAGGAGGGTTTGTCCCGGGCTTGCCTTCGGCGTGGCCTCAGCCCAGTACGTTCTCGCGAATATGCTCTACTGGTTTGACTGGAAACTCCCATCAGGCGGTTCGAAGCTGGCCGAGACTCTGGACATGTCTGAAGTCTACGGCCTCACTGTTCACAAGAAGTCGCCCCTCTACCTGGTTCCGACTCCTTACAGCCCGTGA |
| *rPdUGT94AF1* | ATGTTCTCCGATCAGAGGTCGCTTACGATATTGATGCTCCCCTGGCTCGCGCATGGTCACATTTCTCCGTACCTGGAGTTGGCGAAAAAGCTGACGACCAAACGTAATTTCCACATATTCATCTGCTCTACCCCAGTGAATCTCTCCTCTATAAAGCCGAAGCTCTCACAGAAGTATTCCCATTGTATAGAGTTTGTTGAATTGCACCTTCCTCACGACGACCTCCCCGAGCTTCCTCCTCATTACCACACCACCAATGGGCTTCCTCCGCATCTGATGTCGACCCTGAAAACAGCCTTTGACATGTCTAGTAACAATTTTAGCAACATTCTCAAGACCTTGTCTCCGGATCTCCTTATTTATGATGTGTTGCAGCCTTGGGCCCCGTCCCTCGCCAGCCTTCAGAATTTCCCGTCGATCGAGTTCACTCCTATGGGCGCGGCCCTTACCTCCTTCAGTATTCAGCATATTAAGAATCCTAGTGTCAAGTTTCCATTCCCTTCTATCTATTTGCAAGACTATGAGGCGGAGAAATTCAACAACCTCCTTGAGAGCTCTGCAAACGGGATCAAGGATGGAGACCGCGTGCAGCAGTGTTCAGCGCGGTCCTGTAACATCATTTTGGTGAAGACATCCAGAGAGATTGAGGAGAAATACATCGACTACCTTTCGGATCTGATGGGCAAAAAGATTGTGCCTGTTGGAACTTTGGTTCAAGAACCCATGGATCAGAAGGTCGATGAGGAGACATGGATTATGAAATGGCTGAACAAGATGGAAAGGTCGAGCGTCGTTTACGTCTGTTTCGGATCCGAATATTTCCTGAGCAAGGAGCAAATCGAAGAAATAGCACACGGGCTCGAGCTCTCAAAGGTGAGCTTCATCTGGGTGATACGGTTCCCGAAAGCGGAAAGGAGTACTCGTGTGGAGGAGGTCCTCCCAGAGGGATTCCTCCAGCGTGTCGGCGAAAAGGGGGTCATCATGAAGGGATGGGCTCCGCAGGCGAAGATTTTGCAACACTCATCCGTCGGTGGGTTCGTGAGCCATTGCGGCTGGAATTCTGTGCTCGAGAGCATAAAATTCGGAGTCCCGGTGATAGCGATGCCCATGCATCTCGACCAACCCATCAATGCACGGCTGGTTGAAGAGGTGGGCGTTGGTGTCGAGGTCAAGCGCACAGGCGAGGGCTCCTTGCAGAGGGAGGAGGTGGCTAAGGTGATCCGGGATGTGGTGGTAGAGAAATTCGGCGAAGGCGTGCGCAAGAAAGCGCTGAAGATATCCGACAATGTTAACAAGAAGGAGGACGAGGAGATCGACGGTGTCGTCGAGGAGCTGATCCAGGCCTGTACAGGACGGGGTATTTGA |
| *rPdUGT85A19* | ATGAGCCCTGTGGCGTCTAAAGAAAAGCCTCACGCGGTCTTTGTGCCGTTCCCGGCACAGGGCCATATCAATCCAATGCTGCAATTGGCCAAGTTGCTTAATTATAAGGGCTTTCATATCACCTTCGTCAACACGGAGTTTAATCACAAGAGGATGCTGGAGAGTCAGGGGAGTCATGCGCTCGACGGCCTCCCGTCATTCAGGTTTGAAACCATTCCCGACGGACTCCCGCCTGCCGATGCTGACGCCCGTAGGAACCTCCCGCTCGTATGCGACTCTACTTCCAAGACGTGCCTGGCGCCCTTTGAGGCGCTCTTGACCAAACTGAACTCTAGCCCGGACTCACCTCCAGTGACTTGTATCGTGGCGGATGGTGTGTCCTCCTTCACACTGGATGCGGCAGAGCACTTCGGCATTCCGGAGGTCCTGTTCTGGACCACCAGCGCATGTGGGCTCATGGGCTACGTGCAGTATTACCGTTTGATCGAGAAGGGCCTCACACCCTTCAAGGATGCCAAGGATTTCGCCAATGGTTACCTGGACACAGAGATTGACTGGATTCCGGGGATGAAAGATGTGCGCCTGAAGGACATGCCGTCCTTCATCCGGACTACCGATCCCAACGATATTATGTTGCATTACATGGTGTCCGAGACAGAGCGGAGCAAGAAGGCGTCGGCCATAATCCTGAACACCTTTGACGCCCTCGAGCAGGAAGTGGTTGATGCATTGAGCACCCTCCTCCCGCCAATTTATAGTATTGGACCTCTTCAGCTGCCGTACAGTGAGATCCCGTCCGAGTATAACGATCTGAAAGCCATCGGGAGCAACCTTTGGGCGGAGAATACAGAGTGCCTCAACTGGCTCGATACGAAAGAGCCAAATTCCGTTGTCTACGTGAATTTTGGTTCCACAACCGTAATGACCAACGAACAGCTCGTGGAGTTTTCCTGGGGTTTGGCCAACTCAAAGAAACCTTTTCTCTGGATCATCCGTCCAGGCTTGGTGGCGGGCGAAACTGCCGTCGTCCCGCCGGAGTTTCTGGAGGAAACGAAAGAGAGGGGTATGCTGGCGTCGTGGTGCCCTCAGGAGCAGGTACTCCTCCATTCCGCTATCGGCGGATTCTTGACACACTCTGGCTGGAATTCGACACTGGAGGCCTTGTGCGGCGGTGTCCCTCTTATTTGCTGGCCATTCTTTGCGGAACAGCAGACAAATGTCAGGTACTCGTGCACTCAGTGGGGCATCGGTATAGAGATAGACGGTGAGGTGAAGCGCGACTATATTGACGGCTTGGTGAGGACACTCATGGATGGCGAAGAGGGTAAGAAGATGAGGAAGAAAGCCCTTGAGTGGAAGAAACTCGCAGAGGACGCTACCTCCCCTAAGGGATCAAGTTACCTGGCCCTTGAAAACGTCGTGAGCAAAGTCCTCCTGTCACCAAGGGATTGA |

Start/Stop codons are indicated in red; F2A and P2A encoding sequences are indicated in bule and green, respectively.

**Supplemental Table 2.** Concentrations of key metabolites in the amygdalin biosynthetic pathway in fresh grains from NGZ, ZH11, and transgenic lines.

| Lines | Phenylalanine  (μg·g⁻¹) | (*E*)-Phenylacetaldoxime  (ng·g⁻¹) | Mandelonitrile  (ng·g⁻¹) | Prunasin  (μg·g⁻¹) | Amygdalin  (μg·g⁻¹) |
| --- | --- | --- | --- | --- | --- |
| NGZ | 35.88 ± 0.15 | ND | ND | ND | ND |
| ***C*** | 68.53 ± 15.01 | ND | ND | ND | ND |
| ***CC*** | 100.19 ± 6.12 | ND | 88.19 ± 4.73 | 0.22 ± 0.03 | 0.02 ± 0.00 |
| ***CCU*** | 56.77 ± 0.95 | ND | 28.73 ± 7.12 | 1.83 ± 0.01 | 0.12 ± 0.02 |
| ***CCUU*** | 70.91 ± 0.32 | ND | 69.08 ± 1.09 | 0.61 ± 0.03 | 14.54 ± 0.12 |
| ***CC'UU*** | 48.31 ± 4.48 | ND | 37.13 ± 1.80 | 0.38 ± 0.15 | 0.41 ± 0.05 |
| ***CCUU'*** | 107.12 ± 4.52 | ND | 64.54 ± 3.09 | 0.69 ± 0.01 | 3.41 ± 0.05 |
| ***CCU'U'*** | 92.49 ± 3.63 | ND | 76.87 ± 1.05 | 0.67 ± 0.06 | 3.22 ± 0.01 |
| **ZH11** | 94.63 ± 0.70 | ND | ND | ND | ND |
| ***^j^CCUU*** | 124.40 ± 1.10 | ND | 88.81 ± 1.56 | 0.59 ± 0.01 | 13.17 ± 0.05 |

ND: not detectable.

**Supplemental Table 3.** Proportions of rice weevils selecting NGZ versus transgenic lines.

| Lines | Selection rate (%) | Response rate (%) |
| --- | --- | --- |
| NGZ | 18.29 ± 1.65^b^ | 91.67 ± 3.97 |
| ***C*** | 22.85 ± 1.24^a^ |  |
| ***CC*** | 14.64 ± 0.69^bc^ |  |
| ***CCU*** | 16.21 ± 1.25^bc^ |  |
| ***CCUU*** #1 | 14.46 ± 1.00^bc^ |  |
| ***CCUU*** #2 | 13.56 ± 1.38^c^ |  |

Means (± SE, *n* = 4) with the same letter are not significantly different, as assessed by Duncan's multiple range test (*P* < 0.05).

**Supplemental Table 4.** Proportions of rice weevils selecting ZH11 versus ***^j^CCUU*** rice.

| Lines | Selection rate (%) | Response rate (%) |
| --- | --- | --- |
| ZH11 | 64.55 ± 0.79 | 68.89 ± 3.85 |
| ***^j^CCUU*** | 35.45 ± 0.79*** |  |
| Blank1 | 34.32 ± 2.31 | 74.44 ± 1.93 |
| ZH11 | 65.68 ± 2.31*** |  |
| Blank2 | 47.29 ± 7.93 | 56.67 ± 3.34 |
| ***^j^CCUU*** | 52.71 ± 7.93 |  |

Means with ± SE, *n* = 3; ***, *P* < 0.001.

**Supplemental Table 5.** Developmental metrics measured in rice weevils reared on NGZ and transgenic lines.

| Lines | Eclosion rate (%) | Adult development stage (d) | Weight (mg) | Length (mm) | Width (mm) |
| --- | --- | --- | --- | --- | --- |
| NGZ_rf_ | 42.60 ± 0.11^a^ | 29.57 ± 0.03^a^ | 18.73 ± 0.12^a^ | 2.66 ± 0.03^b^ | 0.89 ± 0.01^ab^ |
| ***C***_rf_ | 38.60 ± 0.50^b^ | 29.13 ± 0.09^b^ | 16.42 ± 0.04^c^ | 2.84 ± 0.03^a^ | 0.85 ± 0.02^bc^ |
| ***CC***_rf_ | 38.71 ± 0.32^b^ | 29.13 ± 0.19^b^ | 14.93 ± 0.12^e^ | 2.86 ± 0.05^a^ | 0.96 ± 0.04^a^ |
| ***CCU***_rf_ | 33.98 ± 0.16^c^ | 28.63 ± 0.07^c^ | 16.97 ± 0.18^b^ | 2.71 ± 0.04^b^ | 0.85 ± 0.03^bc^ |
| ***CCUU*** #1_rf_ | 34.76 ± 0.55^c^ | 28.87 ± 0.18^bc^ | 16.27 ± 0.03^c^ | 2.69 ± 0.06^b^ | 0.82 ± 0.02^bc^ |
| ***CCUU*** #2_rf_ | 34.64 ± 0.49^c^ | 28.80 ± 0.10^bc^ | 15.70 ± 0.06^d^ | 2.70 ± 0.02^b^ | 0.80 ± 0.03^c^ |

Means (± SE, *n* = 10) with the same letter are not significantly different, as assessed by Duncan's multiple range test (*P* < 0.05).

**Supplemental Table 6.** Developmental metrics measured in rice weevils reared on ZH11 and ***^j^CCUU*** rice.

| Lines | Eclosion rate (%) | Adult development stage (d) | Weight (mg) | Length (mm) | Width (mm) |
| --- | --- | --- | --- | --- | --- |
| ZH11_rf_ | 31.72 ± 0.54 | 29.90 ± 0.06 | 20.00 ± 0.17 | 2.62 ± 0.05 | 0.97 ± 0.01 |
| ***^j^CCUU***_rf_ | 20.60 ± 0.36*** | 28.77 ± 0.27* | 13.43 ± 0.09*** | 2.66 ± 0.04 | 0.88 ± 0.00*** |

Means with ± SE, *n* = 10; *, *P* < 0.05; ***, *P* < 0.001.

**Supplemental Table 7.** Oligos used in this study.

| Oligos | Sequence (5′-3′) |
| --- | --- |
| Vector construction | |
| F-PdCYP79D16 | TCCAATAACATCCTCAAATAGCTATGGAAGCTAACGTGGGCTT |
| R-PdCYP79D16 | CCAAGATTTCGAGATCAGGTTCAAGTTTGGTACACGTGCG |
| F-PdCYP71AN24 | CCAATCCAGGACCGGGCTCCATGGCGCTGTTGACCTTGTT |
| R-PdCYP71AN24 | CCAAGATTTCGAGATCAGGTTCAGGGGGAGTAGGGCGTAG |
| F-PdUGT94AF3 | CCATAAGCAAGTACAAATAGCTATGGATAGCAGTCAGCAAAGAAAG |
| R-PdUGT94AF3 | CTTTATTGCCAAATGTTTGAACTCACTCTTTTCTCATACAGAGCTGG |
| F-PdUGT94AF2 | AGAACCCTGGTCCTGGCTCCATGGTGTATTCCGAGCATAA |
| R-PdUGT94AF2 | CTTTATTGCCAAATGTTTGAACTCACTTAAATGTGCTAATTTGTTTCCC |
| F-PmCYP71AN24 | CCAATCCAGGACCGGGCTCCATGGCATTGCTCACGCTCTT |
| R-PmCYP71AN24 | CCAAGATTTCGAGATCAGGTTCACGGGCTGTAAGGAGTCG |
| F-PdUGT85A19 | CCATAAGCAAGTACAAATAGCTATGAGCCCTGTGGCGTCTAAAG |
| R-PdUGT85A19 | GTTAGTAGCGGAGCCATCCCTTGGTGACAGGAGGACT |
| F-PdUGT94AF1 | AGAACCCTGGTCCTGGCTCCATGTTCTCCGATCAGAGGTC |
| R-PdUGT94AF1 | CTTTATTGCCAAATGTTTGAACTCAAATACCCCGTCCTGTAC |
| qRT-PCR analysis | |
| F-/R-PdCYP79D16 RT | TCCACCCTCGGTACTTCCAT / CCAGCCTCGGTCAAGTCTAT |
| F-/R-PdCYP71AN24 RT | GGAGAGTATGTCCAGGCCTT / GTCTCGGCCAATTTTGATCC |
| F-/R-PdUGT94AF3 RT | TGAAGCGCGATGAGATCGCT / GTCCTCCCGCTTCTTCATGT |
| F-/R-PdUGT94AF2 RT | ACGGACCGGAGAGGGAATCT / TATCCCGGAGCTCCAATGCT |
| F-/R-PmCYP71AN24 RT | TGGCCTCAGCCCAGTACGTT / AGGCCGTAGACTTCAGACAT |
| F-/R-PdUGT85A19 RT | CGCGACTATATTGACGGCTT / AGGTAGCGTCCTCTGCGAGT |
| F-/R-PdUGT94AF1 RT | ATCTCGACCAACCCATCAAT / CCGAATTTCTCTACCACCAC |
| F-/R-OsCCR21 RT | GTATCCGATCCCTACAAGGT / AGAACTTTGCCTGTGAAGCT |
| F-/R-OsCCR1 RT | GTACCCTGCCACTGCAAAGT / CATGCATAAGACCGCCTCAT |
| F-/R-Os4CL5 RT | GAGCTCGAGGCCATGCTCAT / AACTTTGCTCTCAGATCCTT |
| F-/R-OsSCPL19 RT | GGCGACCATGATGCTGTTCT / GGCTGAACATTGCAAAACAT |
| F-/R-OsCAD3 RT | TCGTCCACGGGAACAGGACT / GACGTCGATGACGAAGCGAT |
| F-/R-OsPox RT | TCTTCAGACGCCAACCGTGT / TTTGATCCCGTCAACGGTGT |
| F-/R-OsFLS2 RT | GTCAAGCCGTCCAACGTCCT / CACCCCGAAGCTGAACACGT |
| F-/R-OsCPK4 RT | CCAGGCTGCTTTCAGCAAAT / CCTCTTGGACTGGGAAGGTT |
| F-/R-OsCDPK13 RT | AACTGGAGCGAGAGGAACAT / CCCATATTGCCCTTGGTCAT |
| F-/R-OsSTK RT | TGAAGGATGGCTTAAGCCTT / CAGAAACCTGTGACATTCCT |
| F-/R-OsRPM1 RT | GTGAGTAGTGCAAGCAGCTT / CCCAAAAGCCACAGCTTCTT |
| F-/R-OsrbohH RT | GATCGAGCTCCACAACCACT / TGGTTCACGGCGACGCGCTT |
| F-/R-OsCML5 RT | CGGGAGGCGTTCAAGGTGTT / GAACTCGTCGAAGTTGACCT |
| F-/R-OsRPS RT | CACCTGGAGTCGCTCAACCT / TCGCACTCCACCCTCACCTT |
| F-/R-OsASNase1 RT | GCCATCATCCGGCACACGGT / CCGGTGGTGTTGTACGCCAT |
| F-/R-OsASNase2 RT | GGCGGGCTGATGAATAAGAT / CTGAACATGCCGGTGCAGTT |
| F-/R-Os6bglu25 RT | AAGACGTCCTACAAGATGAT / GAACCATTGGACTGATGCTT |
| F-/R-OsActin RT | TCTTCCAGCCTTCCTTCA / ATCCACCTCGCACTTCAT |
| Genotype identification | |
| P-F | GCATCCATTCTCAGGCTGTC |
| P-R1 | CGTTATGTTTATCGGCACTT |
| P-R2 | ACCTTGACCGGTGAGGTAGG |

The underlines highline the sequences that match the vector bone.

**Supplemental Table 8.** Gene accession numbers used in this study.

| Species | Gene name | Accession number |
| --- | --- | --- |
| *Oryza sativa* L. | *OsCCR21* | *LOC_Os02g08420* |
|  | *OsCCR1* | *LOC_Os02g56680* |
|  | *Os4CL5* | *LOC_Os08g34790* |
|  | *OsSCPL19* | *LOC_Os11g24180* |
|  | *OsCAD3* | *LOC_Os10g29470* |
|  | *OsPox* | *LOC_Os12g02080* |
|  | *OsFLS2* | *LOC_Os04g52780* |
|  | *OsCPK4* | *LOC_Os02g03410* |
|  | *OsCDPK13* | *LOC_Os04g49510* |
|  | *OsSTK* | *LOC_Os02g11930* |
|  | *OsRPM1* | *LOC_Os11g12330* |
|  | *OsrbohH* | *LOC_Os12g35610* |
|  | *OsCML5* | *LOC_Os12g41110* |
|  | *OsRPS* | *LOC_Os03g14900* |
|  | *OsASNase1* | *LOC_Os03g40070* |
|  | *OsASNase2* | *LOC_Os04g55710* |
|  | *Os6bglu25* | *LOC_Os06g46940* |
